# Impact of ethnicity on the accuracy of measurements of oxygen saturations: A retrospective observational cohort study

**DOI:** 10.1016/j.eclinm.2022.101428

**Published:** 2022-05-06

**Authors:** Mansoor N. Bangash, James Hodson, Felicity Evison, Jaimin M. Patel, Andrew McD Johnston, Suzy Gallier, Elizabeth Sapey, Dhruv Parekh

**Affiliations:** a Birmingham Acute Care Research Group, Institute of Inflammation and Ageing, New Queen Elizabeth Hospital, University of Birmingham, 1st Floor, Mindelsohn Way, Birmingham B15 2WB, United Kingdom; b Department of Critical Care, University Hospitals Birmingham NHS Foundation Trust, Birmingham, United Kingdom; c Department of Health Informatics, University Hospitals Birmingham NHS Foundation Trust, Birmingham, United Kingdom; d PIONEER: Health Data Research UK (HDRUK) Health Data Research Hub for Acute Care, United Kingdom; e Department of Acute Medicine Acute Medicine, University Hospitals Birmingham NHS Foundation Trust, Birmingham, United Kingdom

**Keywords:** Inequalities, Ethnicity, Oxygen saturations

## Abstract

**Background:**

Pulse oximeters are routinely used in community and hospital settings worldwide as a rapid, non-invasive, and readily available bedside tool to approximate blood oxygenation. Potential racial biases in peripheral oxygen saturation (SpO2) measurements may influence the accuracy of pulse oximetry readings and impact clinical decision making. We aimed to assess whether the accuracy of oxygen saturation measured by SpO2, relative to arterial blood gas (SaO2), varies by ethnicity.

**Methods:**

In this large retrospective observational cohort study covering four NHS Hospitals serving a large urban population in Birmingham, United Kingdom, consecutive pairs of SpO2 and SaO2 measurements taken on the same patient within an interval of less than 20 min were identified from electronic patient records. Where multiple pairs of measurements were recorded in a spell, only the first was included in the analysis. The differences between SpO2 and SaO2 measurements were compared across groups of self-identified ethnicity. These differences were subsequently adjusted for age, sex, bilirubin, systolic blood pressure, carboxyhaemaglobin saturations and the time interval between SpO2 and SaO2 measurements.

**Findings:**

Paired O2 saturation measurements from 16,818 inpatient spells between 1st January 2017 and 18th February 2021 were analysed. The cohort self-identified as being of White (81.2%), Asian (11.7%), Black (4.0%), or Other (3.2%) ethnicities. Across the cohort, SpO2 was statistically significantly higher than SaO2 (*p* < 0.0001), with medians of 98% (interquartile range [IQR]: 95–100%) vs. 97% (IQR: 96–99%), and a median difference of 0.5% points (pps; 95% confidence interval [CI]: 0.5–0.6). However, the size of this difference varied considerably with the magnitude of SaO2, with SpO2 overestimating by a median by 3.8pp (IQR: 0.4, 8.8) for SaO2 values <90% but underestimating by a median of 0.4pp (IQR: -2.0, 1.4) for an SaO2 of 95%. The differences between SpO2 and SaO2 were also found to vary by ethnicity, with this difference being 0.8pp (95% CI: 0.6–1.0, *p* < 0.0001) greater in those of Black vs. White ethnicity. These differences resulted in 8.7% vs. 6.1% of Black vs. White patients who were classified as normoxic on SpO2 actually being hypoxic on the gold standard SaO2 (odds ratio: 1.47, 95% CI: 1.09–1.98, *p* = 0.012).

**Interpretation:**

Pulse oximetry may overestimate O2 saturation, and this is possibly more pronounced in patients of Black ethnicity. Prospective studies are urgently warranted to assess the impact of ethnicity on the accuracy of pulse oximetry, to ensure care is optimised for all.

**Funding:**

PIONEER, the Health Data Research UK (HDR-UK) Health Data Research Hub in acute care.

**Research in Context**

**Evidence before the study**

We searched PubMed for studies of ethnic disparities in oxygen saturations, published up to Sept 31, 2021. We used the search terms ([“Ethnicity” or “Racial”] and [“Oximetry” OR “Saturations” OR “Oxygen”]). We found one large study from United States of America centres reporting paired measures of oxygen saturation by pulse oximetry (SpO2) and arterial blood gas (SaO2) obtained from two cohorts: one comprising 1333 White patients and 276 Black patients and one multi-centre cohort comprising 7342 White patients and 1050 Black patients from 2014 to 2015, finding a consistent difference of 2% across the spectrum of SpO2 measurements in Black compared to White patients. Other studies have been small and in defined disease states, such as in patients about to be extracorporeally oxygenated. Disparities in all ethnic groups and differences at clinically relevant cut-off values of SpO2 remain unconfirmed yet important due to the widespread use of pulse oximetry in clinical decision making for admission and discharge to hospital.

**Added value of the study**

This is the largest cohort study in a diverse cohort in terms of ethnicity, over a significant period, and in a spectrum of acute illness severity and admission reason, providing real-life and internationally translatable data. We highlight differences in the measurement of oxygenation status when using pulse oximeters and arterial blood gas analysers. Discrepancies are largest when oxygen saturations are lowest, where SpO2 consistently overestimates SaO2. Across the range of O2 saturations, the difference between SpO2 and SaO2 is exaggerated in those of Black ethnicity, potentially placing them at most risk of undetected hypoxic events. Similar but smaller effects were observed in those of Asian ethnicity compared to White patients. Despite the complex relationship between SpO2 and SaO2, these differences between ethnicities appeared to persist over the range of O2 saturations.

**Implications of all the available evidence**

Our findings and others suggest that validation of pulse oximeters should focus on non-White racial populations, in order to identify any shortcomings and drive technological refinements. Whilst newer and more accurate devices are being developed, we urge companies and regulatory authorities to provide performance characteristics across ethnicities, and quantify any bias that may be present, so that clinicians can be fully informed in decision-making.

Alt-text: Unlabelled box

## Introduction

Ensuring optimal oxygen delivery to tissues is a prime objective of acute/critical medical care. Using arterial blood gas (ABG) sampling and co-oximetry analysis to measure arterial blood oxygen saturation (SaO2) is considered the gold standard means of assessing blood oxygenation.^1^ However, ABG measurement is an invasive and labour-intensive test. Pulse oximeter measured oxygen saturation (SpO2) is a non-invasive approximation of SaO2. First developed in 1974,^2^ pulse oximeters have been routinely used in both community and hospital settings for the past forty years, and are widely acknowledged to be highly important clinical monitoring devices that improve patient safety.^3^

The measurement of oxygen saturation by pulse oximetry is based upon two principles. The first is that oxyhaemoglobin (O2Hb) and deoxyhaemoglobin (Hb) have different absorption spectra, with O2Hb absorbing greater amounts of infrared light and lower amounts of red light, compared to Hb. The second is that beat-to-beat variation of tissue blood volume produces a light transmission signal which depends only on the pulse characteristics of arterial blood. Pulse oximeters emit two wavelengths of light, red at 660 nm and near-Infrared at 940 nm, from a pair of light-emitting diodes located in one side of the probe, which is usually placed on a finger (over the nailbed). The transmission of the two wavelengths of light through the finger is then detected by a photodiode on the opposite arm of the probe, and the relative amount of red and infrared light absorbed are ultimately used to determine the proportion of Hb bound to oxygen.^4^ Pulse oximetry only detects the oxygen saturation of arterial blood, through subtraction of non-varying, venous (deoxygenated) blood spectra, due to the recognition of fluctuations in absorbed light caused by the cardiac cycle.^5^

The accuracy of pulse oximetry readings is vital. Overestimation of actual SaO2 might lead to clinically relevant hypoxaemia remaining undetected and untreated. Conversely, underestimation of actual SaO2 may result in unnecessary oxygen therapy, with the potential risks of hyperoxaemia,^6^ oxygen wastage and inappropriate clinical decision making, including severity of illness assessment and safe discharge from hospital. The United States Regulatory body which governs approvals for medical devices, the Food and Drug Administration (FDA), requires the accuracy of pulse oximeters to be tested against SaO2, with the root-mean-square difference between the SpO2 and the true SaO2 being within ±2–3%.^7^ There are known conditions where SpO2 readings may not accurately reflect the true SaO2. These include, but are not limited to, methaemoglobinaemia,^8^ severe carbon monoxide poisoning,^9^ hyperbilirubinaemia,^5^ excessive movement, hypotension and hypoperfusion states, severe anaemia, and increased blood glycohaemoglobin.^5^^,^^10^^,^^11^

A recent study has highlighted potential racial biases in SpO2 measurements.^12^ In this study, the frequency of occult hypoxaemia that was not detected by SpO2 was almost three times higher in Black patients, compared to White patients. There have been previous, much smaller studies assessing the impact of skin pigmentation on the accuracy of SpO2 compared to SaO2, but often with discordant results.^13^, ^14^, ^15^

Given the widespread use of SpO2 in clinical decision making worldwide, guidance suggesting narrow therapeutic windows for oxygen therapy,^16^ and care pathways placing more emphasis on early discharge, ambulatory and home monitoring for patient management, any systemic differences between SpO2 and SaO2 could have major health implications. On the other hand, adjustments to measurements on the basis of ethnicity (for example, the estimated glomerular filtration) are now increasingly controversial,^17^^,^^18^ and there has been considerable interest in revalidating scoring systems to remove references to ethnicity.^19^ As such, any changes to clinical practice or test interpretation driven by ethnicity requires a sound evidence base.

The aim of this study was to assess whether SpO2 accurately predicted SaO2, measured by ABG, and whether disparities between ethnicities existed. The study was based in Birmingham, one of the most diverse urban centres in the United Kingdom (UK).^20^

## Methods

### Study design and population

This data study was supported by PIONEER,^21^ a Health Data Research Hub in Acute Care, with ethical approval provided by the East Midlands – Derby REC (reference: 20/EM/0158) and the requirement of informed consent waived. The findings are reported as per the Strengthening the Reporting of Observational Studies in Epidemiology (STROBE) guidelines.^22^

University Hospitals Birmingham NHS Foundation Trust (UHB), UK, is one of the largest hospital complexes in Europe, covering four NHS hospital sites, treating over 2.2 million patients per year, and housing the largest single critical care unit (CCU) in Europe. UHB provides secondary care to a diverse population of 1.3 million in Birmingham and Solihull, and provides a full range of tertiary services to the West Midlands region. UHB runs a fully electronic healthcare record (EHR) (PICS; Birmingham Systems), which has been in place since 1999. Data within PICS is time- and date-stamped to the nearest millisecond, and includes all physiology and ABG analysis outputs.^23^

### Case selection and data collection

Data were retrospectively collected for all measurements of oxygen saturation by oximetry and ABG made during inpatient spells at UHB between 1st January 2017 and 18th February 2021. From these, pairs of measurements (SpO2 and SaO2) on the same patient taken within an interval of less than 20 min were identified. All consecutive measurements meeting these criteria were initially included in the study, to reduce the risk of selection bias as much as possible. In order to minimise the effect of within-spell correlation of outcomes, particularly for those patients with extended lengths of stay, only a single pair of measurements per spell were included in the analysis, namely the first valid pair of measurements after admission. Any cases with an O2 saturation by either measure of <80% (threshold defined a priori) were subsequently excluded, as these were deemed likely to be spurious results (for example, misreported venous blood gas measurements for SaO2).

Patient demographics and clinical data were collected from the EHR and from mandatory data sets within the Hospital Trust. Ethnicity was self-reported by the patient or their family members on admission to hospital, as one of five options: “White”, “Asian”, “Black”, “Mixed” and “Other”. The Mixed group was incorporated into the Other group for analysis.

### O2 saturation measurements

ABG analysers automatically update the EHR, with SaO2 measurements being recorded to one decimal place. No such linkage existed for pulse oximeters; hence SpO2 measurements were collected rounded to the nearest integer, and manually entered into the EHR by health care professionals. For the analyses of differences between the two approaches, calculations were made to one decimal place. However, for analysis of the absolute differences between approaches, the SaO2 values were rounded to the nearest integer, prior to calculations being performed. In addition, the exclusion criteria of O2 saturation <80% used a value of <79.5% for SaO2, for consistency with SpO2.

### Statistical methods

Initially, measurements on SpO2 and SaO2 were compared using Wilcoxon's signed rank test, with the strength of the correlation quantified using Spearman's (rho) coefficient. Since O2 saturations are recorded as percentages, differences between SpO2 and SaO2 were reported as percentage point (pp) differences. These were calculated as SpO2 minus SaO2, such that positive differences represented higher values on SpO2. To test for any proportional bias, the differences between SpO2 and SaO2 were also quantified within subgroups of SaO2 measurements. To model this relationship, a generalized additive model (GAM) was produced, with the pp difference between measures as the dependant variable, and the smooth function of the SaO2 as a continuous covariate. A second model was also produced with the SpO2 as the dependant variable. Since this followed a negatively skewed distribution, SpO2 values were subtracted from 101% to reverse the skew, before being log_2_-transformed, in order to normalise the distribution, and ensure reliability of the model.

SpO2 and SaO2 levels, and the differences between these, were then compared across groups of ethnicity using Kruskal-Wallis tests. The association between ethnicity and the differences between SpO2 and SaO2 was then further interrogated. Since the differences between SpO2 and SaO2 varied by the magnitude of the measurement, the previously described GAM model was extended to additionally include ethnicity as a nominal factor. This model was then further extended to include additional factors, which were selected a priori as variables that were felt to have the potential to influence either O2 saturation, or the accuracy of SpO2 measurements. The goodness of fit was assessed graphically for each factor, with transformations (e.g. log_2_) applied, where poor fit was identified.

Due to the complexities of modelling the relationship between SpO2 and SaO2, an alternative approach was also used as a sensitivity analysis. Here, SaO2 values were dichotomised, into hypoxic (SaO2<94.0%) and normoxic (SaO2≥94.0%), with a threshold of 94.0% being selected a priori, since this is a commonly used clinical oxygenation threshold in acutely unwell patients without chronic respiratory disease.^22^ This was then set as the dependant variable in a binary logistic regression model, with SpO2 as a continuous covariate, and ethnicity as a nominal factor. To further assess the ability for SpO2 to identify hypoxia, the proportions of patients misclassified by SpO2, relative to SaO2, were calculated for each ethnicity, and compared using Chi-square tests.

Continuous variables are reported as (arithmetic) mean ± standard deviation (SD) where approximately normally distributed, or as median (interquartile range; IQR) otherwise. Patients with missing data for ethnicity were excluded from all analyses. Those with missing data for other factors were excluded from the analysis of the affected factor for univariable analysis, whilst multivariable analyses used a complete-cases approach. Analyses were performed using IBM SPSS 24 (IBM Corp. Armonk, NY), with GAM modelling performed using the R package “mgcv”. For each analysis, *p* < 0.05 was deemed to be indicative of statistical significance; no adjustment for multiple comparisons was performed.

### Sample size calculation

After exclusions, data were available for *N* = 16,818 cases, with the differences between SpO2 and SaO2 having a standard deviation of 3.1pp (see *Results* section for further details). Prior to commencing the analysis, a sample size calculation was performed, in order to ensure that there were sufficient data to detect a clinically meaningful difference. Based on the sample size and SD, the study was sufficiently powered to detect a difference in the mean of SpO2 minus SaO2 between patients of White (81.2% of cohort) and Black (4.0%) ethnicity of 0.4pp, at 80% power and 5% alpha.

### Patient and public involvement and engagement (PPIE)

This project was discussed by a multi-ethnic group of patients and members of the public, who considered the background and rationale for the project, the proposed analysis on the basis of self-defined ethnic groups, and the results to date. There was overwhelming support for this work including the use of potentially sensitive data on ethnicity, given the clinical importance and public interest in the topic. This PPIE group will also help write lay summaries of the results to increase public awareness of the outputs.

### Role of the funding source

The funder had no role in the study design, data collection, analysis, or interpretation; the writing of the manuscript or the decision to submit it for publication. All authors confirm they had full access to the study data and accept responsibility to submit the publication and have approved the final version of the manuscript.

## Results

### Cohort characteristics

**Figure 1:**
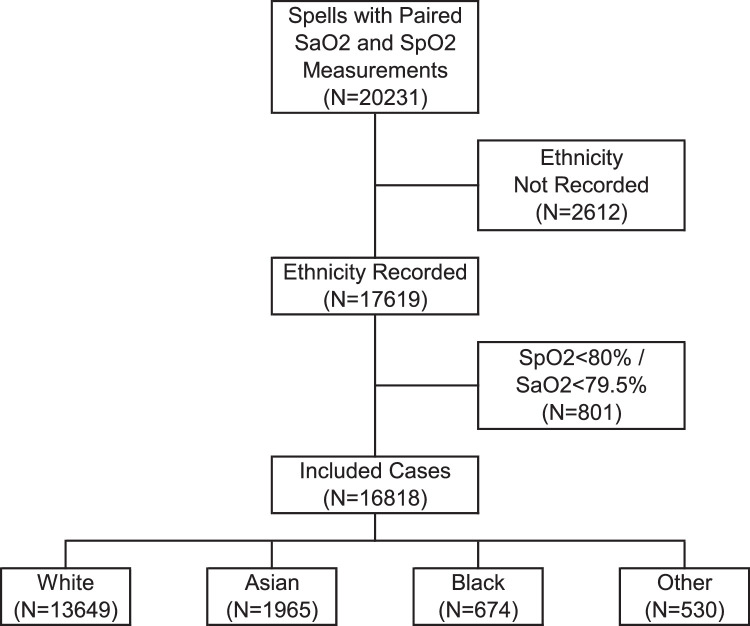
Study flowchart.

Pairs of SpO2 and SaO2 measurements were available for a total of *N* = 20,231 inpatient spells in *N* = 18,069 patients. Of these, the ethnicity was not recorded in *N* = 2612 (12.9%) spells, and these were excluded from further analysis. Of the remaining *N* = 17,619 cases, *N* = 689 (3.9%) had an SaO2<80%, and *N* = 178 (1.0%) had an SpO2<80%; *N* = 66 (0.4%) of these had O2 saturations <80% on both measures. The distributions of O2 saturations <80% were similar across ethnicities (*p* = 0.077, Supplementary Table 1). After excluding these cases, a total of *N* = 16,818 pairs of O2 saturation measurements were included in subsequent analysis (Figure 1).

The median patient age was 63 years (IQR: 50–74), and 57.9% of cases were male. The majority of patients were of self-reported White ethnicity (*N* = 13,649; 81.2%), with the remainder self-reporting Asian (*N* = 1965; 11.7%), Black (*N* = 674; 4.0%) or other ethnicities (*N* = 530; 3.2%; comprising *N* = 222 reporting “mixed” and *N* = 308 reporting “other”). See Table 1 for cohort demography and physiological data.

**Table 1:** Cohort characteristics by ethnicity.

		Total Cohort	Ethnicity			
	N		White (*N* = 13,649)	Asian (*N* = 1965)	Black (*N* = 674)	Other (*N* = 530)
Age (Years)	16,818	63 (50, 74)	65 (53, 75)	57 (42, 68)	55 (45, 68)	50 (35, 64)
*< 50*		4101 (24.4%)	2878 (21.1%)	729 (37.1%)	231 (34.3%)	263 (49.6%)
*50 – 64*		4700 (27.9%)	3740 (27.4%)	578 (29.4%)	243 (36.1%)	139 (26.2%)
*65 – 74*		4045 (24.1%)	3556 (26.1%)	348 (17.7%)	67 (9.9%)	74 (14.0%)
*75 – 84*		2866 (17.0%)	2506 (18.4%)	235 (12.0%)	85 (12.6%)	40 (7.5%)
*85+*		1106 (6.6%)	969 (7.1%)	75 (3.8%)	48 (7.1%)	14 (2.6%)
Sex (% Male)*	16,815	9735 (57.9%)	7796 (57.1%)	1217 (61.9%)	388 (57.6%)	334 (63.0%)
FiO2 (fraction of inspired air)	16,733	0.28 (0.21, 0.40)	0.28 (0.21, 0.40)	0.28 (0.21, 0.45)	0.28 (0.21, 0.40)	0.28 (0.21, 0.45)
PaO2 (kPa)**	16,543	12.7 (10.0, 17.6)	12.6 (10.0, 17.4)	13.4 (10.2, 19.0)	12.9 (10.2, 18.8)	13.0 (10.1, 18.3)
Bilirubin (μmol/L)	16,488	10 (7, 17)	10 (7, 18)	9 (6, 15)	9 (6, 15)	10 (7, 17)
Systolic BP (mmHg)	15,948	124 ± 26	124 ± 26	124 ± 26	132 ± 29	125 ± 26
MetHb (%)	16,680	0.6 (0.5, 0.7)	0.6 (0.5, 0.7)	0.6 (0.5, 0.7)	0.6 (0.5, 0.7)	0.6 (0.5, 0.7)
COHb (%)	16,681	1.4 (1.1, 1.7)	1.4 (1.1, 1.7)	1.2 (0.9, 1.5)	1.3 (1.1, 1.7)	1.3 (1.1, 1.6)
Time SpO2 to SaO2 (Minutes)						
*SaO2 Minus SpO2*	16,818	0.0 (−7.4, 7.8)	0.0 (−7.5, 7.8)	0.0 (−6.9, 8.0)	0.0 (−7.0, 8.1)	0.0 (−8.0, 7.3)
*Absolute Difference*	16,818	7.6 (2.9, 13.0)	7.6 (2.9, 13.0)	7.5 (2.6, 12.9)	7.6 (3.0, 12.8)	7.5 (3.2, 13.1)
O2 Saturation (%)						
*SaO2*	16,818	97.4 (95.6, 98.7)	97.4 (95.6, 98.6)	97.6 (95.6, 98.8)	97.5 (95.7, 98.8)	97.6 (95.6, 98.8)
*SpO2*	16,818	98 (95, 100)	97 (95, 100)	98 (95, 100)	99 (96, 100)	98 (95, 100)
Difference in O2 Saturation (pp)						
*SpO2 Minus SaO2*	16,818	0.5 (−1.2, 1.4)	0.4 (−1.3, 1.4)	0.7 (−0.7, 1.5)	0.8 (−0.3, 1.9)	0.7 (−1.0, 1.6)
Absolute Difference***	16,818	1 (1, 3)	1 (1, 3)	1 (1, 2)	1 (1, 3)	1 (1, 3)

Continuous variables are reported as median (interquartile range) or mean ± standard deviation; nominal variables are reported as N (column%).*vs. female – excludes *N* = 3 who identified as Non-Binary. **Temperature-corrected. *** SaO2 values were rounded to the nearest integer when calculating absolute differences, for consistency with the SpO2 measurements. pp=percentage points; SaO2=arterial O2 saturation; SpO2=O2 saturation on oximetry. FiO2=fraction of inspired oxygen; PaO2=partial pressure of oxygen; BP=blood pressure; MetHb=methaemoglobin; COHb=carboxyhaemoglobin.

### O2 saturations on SpO2 vs. SaO2

**Figure 2:**
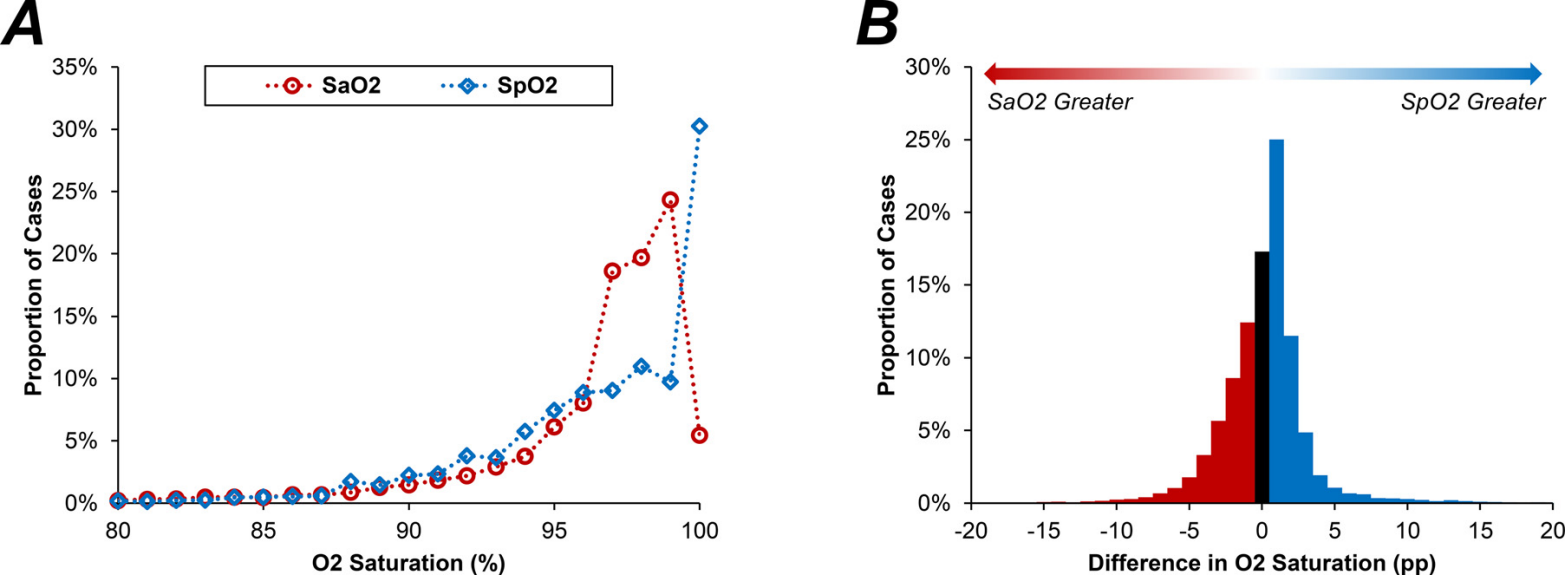
Distributions of SaO2 and SpO2. In Fig. A, points represent the proportion of cases with each integer value of O2 saturation; SaO2 values were rounded to the nearest integer before plotting. Fig. B is a histogram of the percentage point (pp) differences in SpO2 minus SaO2 (with SaO2 values rounded to the nearest integer). Each bar represents 1 pp of O2 saturation, with the black bar representing concordance between SpO2 and SaO2 (i.e. a difference of zero). SaO2=arterial O2 saturation; SpO2=O2 saturation on oximetry.

O2 saturations were found to follow a highly negatively skewed distribution; however, the shape of this distribution varied between measurement methods (Figure 2a). On SpO2, 30.2% (*N* = 5087) of cases had an O2 saturation of 100%, compared to only 5.4% (*N* = 915) of cases on SaO2 (i.e. SaO2≥99.5%). The median SpO2 was 98% (IQR: 95–100%), which was statistically significantly higher than the 97.4% (IQR: 95.6–98.7%) on SaO2 (*p* < 0.0001). The correlation between SpO2 and SaO2 was relatively modest, with Spearman's rho of 0.663.

The differences in O2 saturations between measurement methods (SpO2 minus SaO2) were assessed in further detail. These differences were approximately normally distributed, albeit with long tails, with a median difference of 0.5pp (IQR: −1.2, 1.4; 95% CI: 0.5–0.6) and a mean difference of 0.1 ± 3.1pp (95% CI: 0.1–0.2; Figure 2b). After rounding the SaO2 values to the nearest integer, O2 saturations were concordant on the two measurement approaches in only 17.3% (*N* = 2910) of cases, with SpO2 giving the greater value in 47.6% (*N* = 8008), and SaO2 being greater in 35.1% (*N* = 5900).

**Figure 3:**
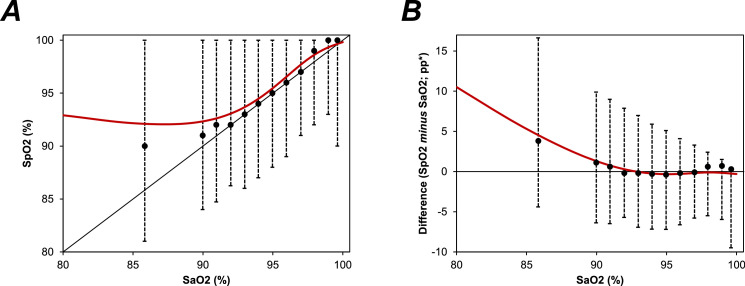
Association between SaO2 and SpO2. Points represent the median SpO2 within intervals of SaO2. The first point includes SaO2 measurements <89.5%, with subsequent intervals having a width of 1% point (89.5–90.4%, 90.5–91.4% etc.). Whiskers represent the 2.5th and 97.5th percentiles, hence the range comprising 95% of values. Red lines are from generalized additive models (GAMs), with the smooth function of SaO2 as the independent variable, and either log_2_(101-SpO2) or the percentage point difference between SpO2 and SaO2 as the dependant variable. *The O2 saturation on SpO2 minus SaO2, as a percentage point (pp) difference. SaO2=arterial O2 saturation; SpO2=O2 saturation on oximetry.

The relationship between SpO2 and SaO2 was then assessed within subgroups of SaO2, in order to test for any proportional bias. This found the difference between measurement methods to vary with the magnitude, following a complex trend, which is visualised in Table 2 and Figure 3. For the lowest values of SaO2 (i.e. <89.5%), SpO2 tended to be greater than SaO2, with a median difference of 3.8pp (IQR: 0.4, 8.8). The size of this difference then reduced with increasing SaO2, with no statistically significant difference between median measurements on SpO2 vs. SaO2 where SaO2 values were in the range 91.5–92.4% (median difference: −0.2; IQR: −2.2, 2.3; *p* = 0.64) or 92.5–93.4% (median difference: −0.2; IQR: −2.0, 1.9; *p* = 0.88). However, SpO2 subsequently began to underestimate, relative to SaO2, with a median difference of −0.4pp (IQR: −2.0, 1.4; *p* = 0.0002) for the subgroup with SaO2 of 94.5–95.4%. The direction of this effect then reversed again, with SpO2 tending to give higher values than SaO2 for the subgroup with SaO2 of 97.5–98.4% (median difference: 0.6pp; IQR: −0.9, 1.8).

**Table 2:** Associations between SaO2 and SpO2.

SaO2 (%)	N Pairs	Difference in O2 Saturation (SpO2 minus SaO2; pp)			O2 Saturation Higher on**:	p-Value
		Mean ± SD	Median (IQR)	95% LoA*		
< 89.5	952	4.7 ± 5.6	3.8 (0.4, 8.8)	−4.4, 16.6	SpO2	<0.0001
89.5 - 90.4	247	1.4 ± 4.0	1.1 (−1.4, 3.9)	−6.4, 9.9	SpO2	<0.0001
90.5 - 91.4	308	0.8 ± 3.8	0.6 (−1.8, 3.1)	−6.5, 9.0	SpO2	0.0018
91.5 - 92.4	369	0.2 ± 3.5	−0.2 (−2.2, 2.3)	−5.7, 7.9	SaO2	0.64
92.5 - 93.4	487	0.0 ± 3.3	−0.2 (−2.0, 1.9)	−6.9, 7.0	SaO2	0.88
93.5 - 94.4	631	−0.4 ± 3.2	−0.3 (−2.2, 1.7)	−7.2, 5.9	SaO2	0.013
94.5 - 95.4	1029	−0.4 ± 3.1	−0.4 (−2.0, 1.4)	−7.2, 5.1	SaO2	0.0002
95.5 - 96.4	1351	−0.4 ± 2.8	−0.2 (−1.8, 1.6)	−6.6, 4.1	SaO2	0.0002
96.5 - 97.4	3128	−0.3 ± 2.5	−0.1 (−1.6, 1.5)	−5.8, 3.3	SaO2	0.021
97.5 - 98.4	3311	0.1 ± 2.2	0.6 (−0.9, 1.8)	−5.5, 2.4	SpO2	<0.0001
98.5 - 99.4	4090	−0.1 ± 2.2	0.7 (−0.5, 1.1)	−6.0, 1.5	SpO2	<0.0001
≥99.5	915	−0.8 ± 2.7	0.3 (−0.6, 0.5)	−9.5, 0.5	SpO2	0.46

Subgroup analyses were performed within intervals of SaO2. For each subgroup, the percentage point (pp) differences between SpO2 and SaO2 are reported as both mean ± standard deviation (SD), and as median (interquartile range; IQR), and compared using Wilcoxon's signed rank test. Bold p-values are significant at *p* < 0.05. *Since the shape of the distribution varied with the magnitude of the measurement, non-parametric 95% limits of agreement (LoA) were used, with the interval defined by the 2.5th-97.5th percentiles. **The measure with the higher O2 saturation, based on the sums of positive vs. negative ranks from the Wilcoxon's test. SaO2=arterial O2 saturation; SpO2=O2 saturation on oximetry; pp=percentage points.

**Figure 4:**
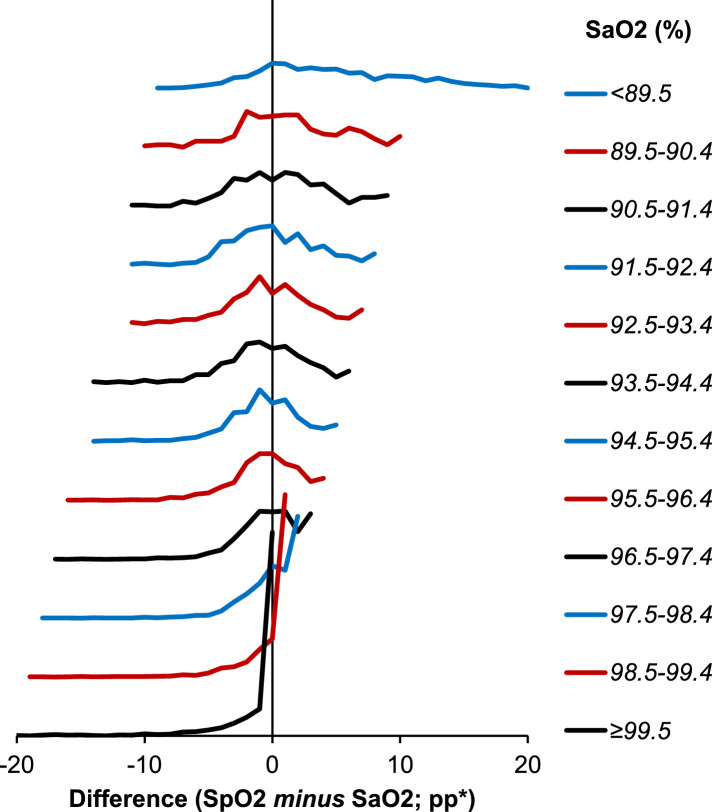
Ridgeline plot of the differences in O2 saturations by SaO2 Lines represent the distributions of the differences in O2 saturations within subgroups of SaO2, and are truncated at the minimum and maximum observed values. *Differences in O2 saturations are calculated as SpO2 minus SaO2, and are reported as percentage point (pp) differences. SaO2=arterial O2 saturation; SpO2=O2 saturation on oximetry.

This subgroup analysis also showed discrepancies between the average difference when quantified as a mean or median. For example, within the subgroup of patients with a SaO2 of 98.5–99.4%, the median difference was positive (0.7pp; IQR: −0.5, 1.1), implying measurements were higher on SpO2, whilst the mean difference was negative (−0.1 ± 2.2 pp), implying measurements were lower on SpO2. This occurred since, whilst the differences between methods was approximately normally distributed for the cohort as a whole (Figure 2b), the distribution became negatively skewed for high O2 saturations, largely since there was an upper bound at an O2 saturation of 100%. This is visualised in the ridgeline plot in Figure 4.

### Cohort characteristics by ethnicity

Details of the cohort characteristics within the four groups of ethnicity are reported in Table 1. White patients had a median age of 65 years (IQR: 53–75), which was considerably greater than the medians of 50–57 years in the three non-White ethnic groups. Bilirubin levels tended to be higher in White patients (median: of 10 μmol/L vs. 9 μmol/L in both the Asian and Black groups), whilst Black patients tended to have higher systolic BP (mean: 132 vs. 124–125 in the remaining groups). However, the time intervals between SpO2 and SaO2 measurements were similar across the ethnicities, with the median absolute differences ranging from 7.5 to 7.6 min across the four groups.

### Differences in O2 saturations by ethnicity

Whilst SaO2 was found to differ statistically significantly between groups (*p* = 0.0010), the magnitude of this difference was relatively small, with medians ranging from 97.4% (IQR: 95.6–98.6%) in White patients to 97.6% (IQR: 95.6–98.8%) in the Asian group. However, the difference in SpO2 was more pronounced, with medians ranging from 97% (IQR: 95–100%) in White patients, to 99% (IQR: 96–100%) in Black patients (*p* < 0.0001). As such, the differences between SpO2 and SaO2 varied statistically significantly with ethnicity, with SpO2 being a median of 0.4pp (IQR: −1.3, 1.4) higher than SaO2 in White patients, compared to 0.8pp (IQR: −0.3, 1.9) higher in those of Black ethnicity (*p* < 0.0001, Table 1).

**Figure 5:**
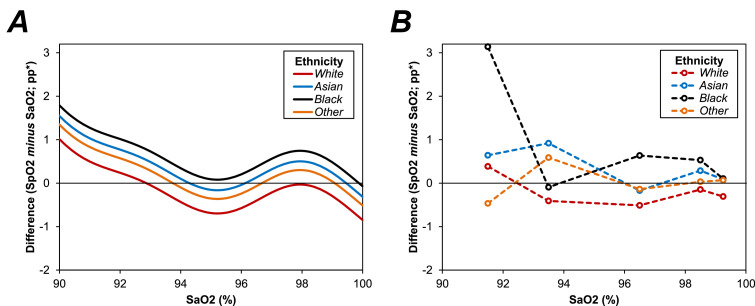
Association between SaO2 and SpO2 by ethnicity. Fig. A represents a generalized additive model (GAM), with the percentage point (pp) difference in O2 saturations between SpO2 and SaO2 as the dependant variable, the smooth function of SaO2 as a covariate, and ethnicity as a nominal factor. Fig. B visualises the observed mean differences between SpO2 and SaO2 within intervals of SaO2, to validate the goodness of fit of the GAM model. Each interval had a width of 2pp, and points are plotted at the midpoint of the interval. Both plots are left-truncated at an SaO2 of 90%, in order to better visualise the differences between groups. *Differences in O2 saturations are calculated as SpO2 minus SaO2, and are reported as percentage point (pp) differences. SaO2=arterial O2 saturation; SpO2=O2 saturation on oximetry.

Since the difference between SpO2 and SaO2 had previously been shown to vary by the magnitude of the measurement, analyses were then performed to compare this between ethnicities, after adjusting for magnitude of SaO2. The resulting model (Figure 5*a*) found the difference between ethnicities to persist, after adjusting for the magnitude of the measurement (*p* < 0.0001). Relative to patients of White ethnicity, the differences between SpO2 and SaO2 were 0.5pp (95% CI: 0.4–0.7; *p* < 0.0001) greater in Asian patients, 0.8pp (0.6–1.0; *p* < 0.0001) greater in Black patients, and 0.3pp (0.1–0.6, *p* = 0.0064) greater in those other ethnicities.

This model was then further extended, to adjust for other potentially confounding factors (Table 3). This found the difference in O2 saturations (SpO2 minus SaO2) to be statistically significantly more positive in males (*p* < 0.0005), younger patients (*p* < 0.0001), those with lower COHb (*p* < 0.0001), and those with higher systolic BP (*p* = 0.041). After adjusting for these factors, the differences between ethnicities remained statistically significant (*p* < 0.0001), with more positive differences between measures of O2 saturations observed for Asian (0.3pp, 95% CI: 0.2–0.5, *p* < 0.0001) and Black (0.6pp, 95% CI: 0.4–0.8, *p* < 0.0001) patients, relative to those of White ethnicity.

**Table 3:** Associations with percentage point differences in O2 saturation between SpO2 and SaO2.

	Analysis 1) Univariable		Analysis 2) SaO2 + Ethnicity		Analysis 3) All Factors	
	Coefficient (95% CI)	p-Value	Coefficient (95% CI)	p-Value	Coefficient (95% CI)	p-Value
SaO2	N/A*	<0.0001	N/A*	<0.0001	N/A*	<0.0001
Ethnicity		<0.0001		<0.0001		<0.0001
*White*	–	–	–	–	–	–
*Asian*	0.49 (0.34, 0.63)	<0.0001	0.53 (0.40, 0.67)	<0.0001	0.33 (0.19, 0.47)	<0.0001
*Black*	0.83 (0.59, 1.07)	<0.0001	0.78 (0.56, 0.99)	<0.0001	0.62 (0.40, 0.84)	<0.0001
*Other*	0.33 (0.07, 0.60)	0.015	0.33 (0.09, 0.57)	0.0064	0.11 (−0.14, 0.35)	0.39
Age (per Decade)	−0.12 (−0.15, −0.09)	<0.0001			−0.20 (−0.22, −0.17)	<0.0001
Sex (Male)	0.03 (−0.06, 0.13)	0.51			0.15 (0.07, 0.24)	<0.0005
Bilirubin (per Two-Fold Increase)**	−0.05 (−0.08, −0.01)	0.015			0.01 (−0.03, 0.04)	0.63
Systolic BP (per 10 mmHg)	0.03 (0.01, 0.05)	<0.0007			0.02 (0.00, 0.03)	0.041
COHb (per 10pp)	−1.14 (−1.70, −0.59)	<0.0001			−3.11 (−3.64, −2.59)	<0.0001
Time from SpO2 to SaO2 (per 10 min)	−0.03 (−0.08, 0.01)	0.17			−0.01 (−0.05, 0.04)	0.75

Results are reported for three analyses, each of which had the percentage point (pp) difference between SpO2 and SaO2 as the dependant variable, with positive values implying a higher measurement on SpO2. Coefficients represent the change in this difference per the stated number of units increase in continuous variables, or for the stated category relative to the reference category for nominal variables. In Analysis (1), a separate univariable model was produced for each factor, with a generalized additive model (GAM) used for SaO2, and general linear models used for the other factors considered. Analysis (2) then extended the GAM of SaO2 to include ethnicity, whilst Analysis (3) further extended this model to include all factors reported in the table. For Analysis (3), only the SaO2 was modelled based on a smooth function, as goodness of fit testing indicated that linear models yielded a reasonable fit for the other factors considered. The numbers of cases included in each model in Analysis (1), after excluding those with missing data, are as per the N's in Table 1. Analysis (3) used a complete-cases approach, and was based on *N* = 15,494 after exclusions. Bold p-values are significant at *p* < 0.05. *Since SaO2 was modelled as a smooth function in the GAM, it was not possible to summarise this trend using a single coefficient; this is instead visualised in Figure 3b for Analysis (1) and Figure 5a for Analysis 2. **Since bilirubin followed a highly skewed distribution, and had a non-linear association with the dependant variable, values were log_2_-transformed, prior to analysis; hence the coefficient represents the change in the outcome per two-fold increase in bilirubin levels. SaO2=arterial O2 saturation; SpO2=O2 saturation on oximetry; COHb=carboxyhaemoglobin; BP=blood pressure; pp=percentage point.

### Associations between SpO2 and normoxia by ethnicity

**Figure 6:**
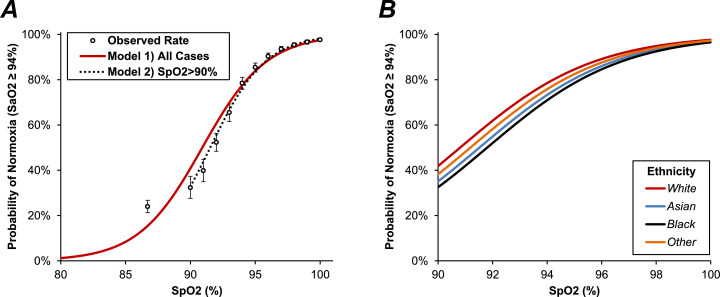
Association between SpO2 and the probability of normoxia (SaO2 ≥ 94.0%) In Fig. A, points represent the probability of SaO2 ≥ 94.0% for SpO2 of 80–89% (first point), and for each integer value of SpO2 from 90 to 100%. Whiskers represent 95% confidence intervals. The red line is from a binary logistic regression model, with SpO2 as a continuous covariate. This model appeared to have suboptimal fit in for low values of SpO2, due to the greater inaccuracy of oximetry in this range. As such, a second model (broken line) was produced which only included the subset with SpO2>90%, which returned similar results in this range. In Fig. B, the first binary logistic regression model was extended to include ethnicity as a factor, see Table 4 for further details; this figure is truncated at an SpO2 of 90%, to more clearly highlight the difference between groups. SaO2=arterial O2 saturation; SpO2=O2 saturation on oximetry.

Due to the complexities of modelling the relationship between SpO2 and SaO2, the analysis of ethnicity was repeated using an alternative approach as a sensitivity analysis. Initially, the SaO2 values were dichotomised using a cut-off value of 94.0%, with the *N* = 14,165 (84.2%) of cases that were greater than or equal to this threshold being classified as normoxic, and the remainder as hypoxic. A binary logistic regression model was then produced, to assess the relationship between SpO2 and normoxia on SaO2, which returned a C-statistic of 0.87 (95% CI: 0.86–0.88; Figure 6a).

This model was then extended to include ethnicity as a factor (Figure 6b, Tables 4, 5), which was found to be statistically significant (*p* < 0.0002). For a given SpO2 value, both Asian (odds ratio [OR]: 0.75, 95% CI: 0.64–0.88, *p* < 0.0004) and Black (OR: 0.67, 95% CI: 0.51–0.87, *p* = 0.0031) patients were statistically significantly less likely to be normoxic, compared to those of White ethnicity, implying a greater tendency for SpO2 to overestimate O2 saturations in these non-White groups

**Table 4:** Binary logistic regression model of normoxia (SaO2 ≥ 94.0%).

	Odds Ratio (95% CI)	*p*-Value
SpO2 (per pp)	1.50 (1.48 - 1.53)	<0.0001
Ethnicity		<0.0002
*White*	–	–
*Asian*	0.75 (0.64 - 0.88)	<0.0004
*Black*	0.67 (0.51 - 0.87)	0.0031
*Other*	0.86 (0.63 - 1.16)	0.32

Results are from a binary logistic regression model, with SaO2 ≥ 94.0% as the dependant variable. SpO2 was included as a continuous covariate, alongside ethnicity. The model was based on *N* = 16,818 (*N* = 14,165 events). Bold p-values are significant at *p* < 0.05. pp=percentage point. SaO2=arterial O2 saturation; SpO2=O2 saturation on oximetry.

**Table 5:** Association between SpO2 and rates of normoxia (SaO2 ≥ 94.0%) by ethnicity.

SpO2	Cases With SaO2 ≥ 94.0%			
	White	Asian	Black	Other
<90%	23.4% (20.6–26.4%) [197/843]	29.6% (20.0–40.8%) [24/81]	24.2% (11.1–42.3%) [8/33]	25.0% (12.7–41.2%) [10/40]
90%	31.8% (26.7–37.3%) [100/314]	31.3% (16.1–50.0%) [10/32]	29.4% (10.3–56.0%) [5/17]	54.5% (23.4–83.3%) [6/11]
91%	41.1% (35.8–46.6%) [139/338]	21.4% (10.3–36.8%) [9/42]	70.0% (34.8–93.3%) [7/10]	50.0% (6.8–93.2%) [2/4]
92%	53.2% (48.9–57.6%) [280/526]	49.4% (38.1–60.7%) [40/81]	40.0% (16.3–67.7%) [6/15]	50.0% (23.0–77.0%) [7/14]
93%	65.4% (61.1–69.5%) [334/511]	63.8% (51.3–75.0%) [44/69]	68.8% (41.3–89.0%) [11/16]	75.0% (47.6–92.7%) [12/16]
94%	78.8% (75.9–81.6%) [651/826]	76.6% (66.7–84.7%) [72/94]	75.0% (53.3–90.2%) [18/24]	78.3% (56.3–92.5%) [18/23]
95%	86.9% (84.7–88.9%) [936/1077]	80.0% (71.3–87.0%) [88/110]	72.7% (54.5–86.7%) [24/33]	69.7% (51.3–84.4%) [23/33]
96%	91.0% (89.3–92.5%) [1140/1253]	86.7% (80.0–91.8%) [124/143]	89.4% (76.9–96.5%) [42/47]	86.0% (73.3–94.2%) [43/50]
97%	94.7% (93.3–95.9%) [1183/1249]	87.6% (81.9–92.1%) [156/178]	88.5% (76.6–95.6%) [46/52]	90.5% (77.4–97.3%) [38/42]
98%	95.7% (94.5–96.6%) [1474/1541]	94.9% (90.8–97.5%) [186/196]	92.3% (83.0–97.5%) [60/65]	93.5% (82.1–98.6%) [43/46]
99%	97.1% (96.0–97.9%) [1288/1327]	95.8% (91.9–98.2%) [183/191]	93.7% (85.8–97.9%) [74/79]	94.9% (82.7–99.4%) [37/39]
100%	97.9% (97.4–98.4%) [3765/3844]	97.2% (95.7–98.3%) [727/748]	94.7% (91.4–97.0%) [268/283]	97.6% (94.6–99.2%) [207/212]

Data were initially divided into subgroups, based on the value of SpO2. Within each subgroup, the proportion of patients with SaO2 ≥ 94.0% is then reported for each ethnicity. Data are reported as: “percentage (95% confidence interval) [n/N]”. SaO2=arterial O2 saturation; SpO2=O2 saturation on oximetry.

In order to further quantify this effect, a misclassification analysis was performed (Table 6). For the cohort as a whole, 41.5% (1251/3013) of those with SpO2<94% had been misclassified, and were actually normoxic on SaO2; this rate was similar across the subgroups of ethnicities (*p* = 0.91). In those with SpO2 ≥ 94%, a total of 6.5% (891/13,805) had been misclassified, and were hypoxic on SaO2. This rate was found to differ statistically significantly with ethnicity (*p* = 0.012), with SpO2 misclassifying 8.7% (51/583) of Black patients as normoxic, compared to 6.1% (680/11,117) of White patients (OR: 1.47, 95% CI: 1.09–1.98).

**Table 6:** Misclassification analysis.

		Hypoxia	Normoxia	Predictive Accuracy (Outcome=Normoxia)	
Subgroup	SpO2	*(SaO2 < 94.0%)*	*(SaO2 ≥ 94.0%)*		
Overall	<94%	1762 (58.5%)	1251 (41.5%)	Sens = 91.2% (90.7–91.6%)	NPV = 58.5% (56.7–60.2%)
	≥94%	891 (6.5%)	12,914 (93.5%)	Spec = 66.4% (64.6–68.2%)	PPV = 93.5% (93.1–94.0%)
*By Ethnicity*					
White	<94%	1482 (58.5%)	1050 (41.5%)	Sens = 90.9% (90.3–91.4%)	NPV = 58.5% (56.6–60.5%)
	≥94%	680 (6.1%)	10,437 (93.9%)	Spec = 68.5% (66.5–70.5%)	PPV = 93.9% (93.4–94.3%)
Asian	<94%	178 (58.4%)	127 (41.6%)	Sens = 92.4% (91.0–93.6%)	NPV = 58.4% (52.6–64.0%)
	≥94%	124 (7.5%)	1536 (92.5%)	Spec = 58.9% (53.2–64.5%)	PPV = 92.5% (91.2–93.7%)
Black	<94%	54 (59.3%)	37 (40.7%)	Sens = 93.5% (91.1–95.4%)	NPV = 59.3% (48.5–69.5%)
	≥94%	51 (8.7%)	532 (91.3%)	Spec = 51.4% (41.5–61.3%)	PPV = 91.3% (88.7–93.4%)
Other	<94%	48 (56.5%)	37 (43.5%)	Sens = 91.7% (88.7–94.1%)	NPV = 56.5% (45.3–67.2%)
	≥94%	36 (8.1%)	409 (91.9%)	Spec = 57.1% (45.9–67.9%)	PPV = 91.9% (89.0–94.3%)

Cases were initially classified as hypoxic or normoxic, based on SaO2. The distribution of hypoxia vs. normoxia was then calculated within those cases with SpO2 <94% and ≥94% for the cohort as a whole, as well as within each subgroup of ethnicity; data are reported as N (row%). The sensitivity (“Sens”), specificity (“Spec”), and positive/negative predictive values (PPV/NPV) of SpO2 for the identification of normoxia on SaO2 were then calculated for each subgroup, and reported with the 95% confidence interval.

## Discussion

This study reports paired oxygen saturation measurements from pulse oximetry and ABG (the accepted gold standard) from almost 17,000 inpatient spells at a secondary care NHS trust with four hospitals, which serves a large diverse urban catchment area. This is the largest study conducted to assess this issue to date, and the first to assess inaccuracy in a large subgroup of Asian patients.

The correlation between SpO2 and SaO2 measurements was found to be relatively modest. This was a result of a complex relationship between SpO2 and SaO2 measurements, the concordance between which varied across the spectrum of O2 saturations considered in this study (80%–100%). For lower SaO2 values, particularly those <90%, SpO2 considerably overestimated oxygenation (relative to SaO2). Above this level, the SpO2 and SaO2 became more consistent, although statistically significant differences were still observed, the direction and magnitude of which changed with the magnitude of the measurement. As such, for the cohort as a whole, the median difference between SpO2 and SaO2 was 0.5pp. This is likely a clinically significant difference, particularly for patients with O2 saturations on the borderline of treatment thresholds, where an error in SpO2 measurements of this size may be sufficient to lead to misclassification, leading to under- or overtreatment. This was visualised in the misclassification analysis, with classifications of hypoxia on pulse oximetry only being concordant with ABG in 58.5% of cases, with correct classification of normoxia in 93.5% of cases.

After adjusting for potentially confounding factors, the differences between SpO2 and SaO2 were found to vary with ethnicity, with pulse oximetry tending to give greater SpO2 measurements in patients of Black compared to White self-reported ethnicity. Similar (albeit smaller) effects were observed in those of Asian ethnicity, compared to White patients. The possible clinical relevance of these differences were clearly visualised in the misclassification analysis. In patients of White ethnicity, 6.1% of those classified as normoxic on pulse oximetry were found to be hypoxic when assessed on ABG. However, the tendency for SpO2 measurements to be higher in patients of Black ethnicity resulted in misclassified hypoxia rate of 8.7%. This finding was consistent with a recent study, which reported falsely reassuring pulse oximetry values in profoundly hypoxic patients, particularly those of Black ethnicity.^24^

Our results are also consistent with a much smaller study of 11 healthy people with darkly pigmented skin and 10 healthy people with light skin pigmentation, who were oxygen restricted as part of a physiological study. This study tested three different pulse oximeters, all of which gave higher readings, relative to SaO2, in those with dark- vs. light-pigmented skin.^25^ A larger follow-on study of 36 participant replicated these findings and, importantly, highlighted differences in performance of devices and variations amongst differing levels of skin pigmentation (dark, light and intermediate). This was consistent with our findings that the magnitude of the discrepancies between SpO2 and SaO2 in Asian patients was in between those of White and Black ethnicities. Our results are also consistent with results from a large study reported in 2020.^12^ They reported paired measures of oxygen saturation by pulse oximetry and ABG obtained from two cohorts: one comprising 1333 White patients and 276 Black patients from 2020, and one multi-centre cohort comprising 7342 White patients and 1050 Black patients from 2014 to 2015, finding a consistent difference of 2% across the spectrum of SpO2 measurements in Black compared to White patients.

Although pulse oximetry has led to huge advances in patient safety through allowing the real-time non-invasive monitoring of oxygen saturation, the findings of the present study suggest that performance characteristics of pulse oximetry across varying levels of saturation, and particularly the differences across races and ethnicities, need to be further assessed. Further studies performing device validation against the gold standard are an urgent priority, in order to improve reliability and prevent unrecognised hypoxia, particularly from the perspective of those parts of the world where oxygen sensitive blood disorders such as sickle cell disease are much more prevalent. This is particularly important as endeavours continue to increase the availability of affordable pulse oximetry across the developing world.

The findings of this study are also pertinent in light of the COVID-19 pandemic, which has disproportionately impacted people due to their race and ethnicity.^26^ This health disparity reflects long-standing fundamental socio-economic inequalities, which not only help to explain higher rates of underlying poor health, but also other factors associated with increased risks of catching and then becoming seriously ill and dying from COVID-19 and other diseases.^27^^,^^28^ There is an increasing drive for early discharges from hospital, as well as for remote home monitoring to avoid unnecessary admissions.^29^^,^^30^ Pulse oximetry is used routinely in all healthcare settings, and is the mainstay of rapid quantification of a patient's oxygenation status worldwide. Although O2 saturations measured on pulse oximetry should never be used in isolation for clinical decision-making, they are included in many severity scoring systems and decision-making algorithms, such as those for pneumonia and COPD. Our findings highlight the need for some caution, as there is potential risk of overestimation of SpO2 measurement, which appears to be particularly pronounced in patients in ethnicities with a tendency for darker skin pigmentation. This could potentially lead to inadequate treatment if, for example, insufficient oxygenation in patients COVID-19 is missed, leading to patients being sent home, rather than admitted, potentially delaying, or denying access to evidence-based therapies.

Strengths of our study include its size and representation of a multi-cultural, urban population in the UK across four hospital sites. The primary limitation of this study was its retrospective nature, with the baseline cohort comprising patients who had undergone assessment of O2 saturation by both oximetry and ABG, which may not be representative of the inpatient cohort as a whole. In addition, initial assessment of the data identified O2 saturations that were unfeasibly low, and likely to represent either erroneous or incorrectly recorded measurements. In an attempt to exclude these spurious values, a minimum O2 saturation of 80% on both oximetry and ABG was selected as a threshold, with measurements below this being excluded. However, if this approach is too strict, then genuine measurements may have been excluded, introducing selection bias, whereas if it was too lenient, then erroneous data may have been included in the analysis.

A second limitation of the study is the potential for confounding, due to the differences in baseline characteristics between the ethnicity groups, for example, the large difference in average age. This age discrepancy has been described in other studies of hospitalised patients where ethnicity was a focus^23^ and, therefore, may reflect true differences in admitted populations, but this could impact on the results of the current study. Whilst multivariable analyses were employed to adjust for these differences, modelling will always leave some degree of residual confounding. There is also the potential for other intangible or unmeasured confounding factors to have influenced the findings of the analysis. Thirdly, the SpO2 and SaO2 readings were separated by on average 7.5 min and were not all simultaneous readings. There is a degree of SpO2 dynamic variation, especially in acutely ill patients that this study design could not account for. Fourthly, the self-reported groups of ethnicity used in the analysis were reasonably broad. As such, whilst these may act as a surrogate of skin colour, there is likely to be considerably within-group variability. If the observed discrepancies in oximetry measurements, relative to SaO2 are, to some degree, a consequence of the degree of skin pigmentation, then this may have acted as a confounding factor in the analysis. Finally, the data included in the study comprised inpatient spells from hospitals based in the same geographical area and operated under the same NHS trust. As such, the results may not be generalizable to other hospitals, particularly if there are large differences in patient demographics.

This study highlights potential differences in the measurement of oxygenation status when using pulse oximeters and ABG. Discrepancies appear to be largest when oxygen saturations are lowest, where SpO2 was seen to consistently overestimate SaO2. Across the range of O2 saturations, the difference between SpO2 and SaO2 is appeared to be exaggerated in those of Black ethnicity, potentially placing them at most risk of undetected hypoxic events. Whilst there is insufficient evidence to change current practice, caution may need to be exercised in some small, specific subgroups of patients with borderline SpO2 levels. Prospective studies are urgently warranted to further quantify the degree of any such racial bias in current devices, to ensure care is optimised for all.

## Declaration of interests

DP reports funding from the NIHR and MRC. ES reports funding support from HDR-UK, MRC, Wellcome Trust, NIHR, Alpha-1-Foundation and British Lung Foundation. ES declares receiving consulting fees from Boehringer Ingleheim and support to attend scientific meetings from Astra Zeneca. MB reports funding from the UK Intensive Care Society. All other authors declare no competing interests

## Appendix Supplementary materials

Image, application 1
