# Supplementary material for: Impact of ethnicity on the accuracy of measurements of oxygen saturations: A retrospective observational cohort study
Source: eClinicalMedicine. 2022 May 6;48:101428. doi: 10.1016/j.eclinm.2022.101428 (PMC9096912; doi:10.1016/j.eclinm.2022.101428)
Supplement: Supplementary file 1 [file mmc1.pdf]

Caption for Supplementary File 1

Supplementary Table 1

Exclusions by ethnicity due to a SpO2 or SaO2 <80%

Supplementary Table 1 – Exclusions by ethnicity

**O2 Saturation**		**Ethnicity**			
SpO2	***SaO2****	***White (N=14300)***	***Asian (N=2049)***	***Black (N=723)***	***Other (N=547)***
≥80%	≥79.5%	13649 (95.4%)	1965 (95.9%)	674 (93.2%)	530 (96.9%)
<80%	≥79.5%	92 (0.6%)	12 (0.6%)	6 (0.8%)	2 (0.4%)
≥80%	<79.5%	501 (3.5%)	66 (3.2%)	41 (5.7%)	15 (2.7%)
<80%	<79.5%	58 (0.4%)	6 (0.3%)	2 (0.3%)	0 (0.0%)

*Chi-square test: p=0.077. *A cut-off value of 79.5%, rather than 80% was used for ABG, since values were reported to one decimal place. SaO2=arterial O2 saturation; SpO2=O2 saturation on oximetry.*
